# Regulation of adult neurogenesis: the crucial role of astrocytic mitochondria

**DOI:** 10.3389/fnmol.2024.1516119

**Published:** 2024-11-22

**Authors:** Danping Liu, Pei Guo, Yi Wang, Weihong Li

**Affiliations:** Basic Medical College, Chengdu University of Traditional Chinese Medicine, Chengdu, China

**Keywords:** astrocytes, mitochondrial, neurogenesis, Alzheimer’s disease, major depressive disorder

## Abstract

Neurogenesis has emerged as a promising therapeutic approach for central nervous system disorders. The role of neuronal mitochondria in neurogenesis is well-studied, however, recent evidence underscores the critical role of astrocytic mitochondrial function in regulating neurogenesis and the underlying mechanisms remain incompletely understood. This review highlights the regulatory effects of astrocyte mitochondria on neurogenesis, focusing on metabolic support, calcium homeostasis, and the secretion of neurotrophic factors. The effect of astrocytic mitochondrial dysfunction in the pathophysiology and treatment strategies of Alzheimer’s disease and depression is discussed. Greater attention is needed to investigate the mitochondrial autophagy, dynamics, biogenesis, and energy metabolism in neurogenesis. Targeting astrocyte mitochondria presents a potential therapeutic strategy for enhancing neural regeneration.

## 1 Introduction

Early childhood experiences, particularly sensory stimulation, are critical for brain development and are intricately linked to neurogenesis, the ongoing generation of new neurons throughout life (Nardou et al., 2019). Neurogenesis, an energy-intensive process, relies heavily on mitochondrial function for adequate adenosine triphosphate (ATP) provision. Consequently, alterations in mitochondrial function can directly impact the efficiency and fidelity of neurogenesis (Khacho et al., 2017).

The brain is primarily composed of neurons and glial cells (Kuramoto et al., 2022). Notably, neuronal and glial mitochondria differentially contribute to neurogenesis. Neuronal mitochondria predominantly provide direct energy supply (Xu et al., 2023), regulate synaptic plasticity (Kochan et al., 2024), etc. Conversely, glial mitochondria primarily offer metabolic support (Allen and Barres, 2009), facilitate myelin sheath protection and repair (Bradl and Lassmann, 2010), and participate in immunomodulation (Morrison et al., 2023). While the impact of neuronal mitochondria on neurogenesis has been extensively investigated (Iwata et al., 2020; Dario et al., 2021; Ozgen et al., 2022), the contribution of glial cell mitochondria, particularly those within astrocytes (the most abundant glial cell type in the brain), remains relatively underexplored.

Neurogenesis exhibits distinct characteristics across different developmental stages, varying in rate, location, function, and the involvement of astrocytic mitochondria. During fetal development, rapid and widespread neurogenesis establishes the fundamental brain architecture. Under physiological conditions, astrocytic mitochondria provide metabolic support and energy, regulating neuronal migration and circuit formation. Mitochondrial dysfunction during this period (e.g., due to maternal infection) can lead to neurodevelopmental abnormalities (Kostović et al., 2019; Vasistha et al., 2020). In infancy and early childhood, neurogenesis slows and becomes localized to specific brain regions, supporting circuit refinement and functional maturation. Astrocytic mitochondria contribute to synaptogenesis and myelination, maintaining neuronal homeostasis. Dysfunction at this stage (e.g., due to brain injury) can disrupt circuit development (Bosworth and Allen, 2017; Ortiz-González, 2021). In adulthood, neurogenesis is primarily associated with learning, memory, and mood regulation. Astrocytic mitochondria support the maintenance of neural stem cell niches and synaptic plasticity. Dysfunction in adulthood (e.g., from chronic stress) can contribute to cognitive decline and neurological disease (Du et al., 2018; Morita et al., 2019).

Given the critical role of astrocytic mitochondria in neurogenesis and the significant research implications, this review focuses on their contribution to adult neurogenesis. We explore how astrocytic mitochondrial dysfunction impacts adult neurogenesis and its connection to neurological disorders, aiming to provide novel insights and therapeutic strategies for these conditions.

Despite evidence supporting adult neurogenesis in mammals since the 1960s (Altman, 1962; Altman and Das, 1965; Kaplan and Hinds, 1977), its frequency (Sorrells et al., 2018) and functional significance (Kempermann, 2012) remain debated. Neurogenesis, the process by which neural stem cells (NSCs) differentiate into mature neurons (Gage, 2019), encompasses NSC proliferation and differentiation, neuronal precursor cell migration and differentiation, and ultimately, the integration of newborn neurons into existing neural circuits (Ming and Song, 2011). In the adult mammalian brain, two primary neurogenic niches harboring endogenous NSCs are the subventricular zone (SVZ) lining the lateral ventricles (Alvarez-Buylla and Garcia-Verdugo, 2002) and the subgranular zone (SGZ) of the hippocampal dentate gyrus (Gage, 2000). Recent research has also highlighted the potential for neurogenesis in the neocortex (Zamboni et al., 2020). Neurogenesis plays a critical role in brain development and sculpting (Semple et al., 2013) and the formation of neural circuits (Obernier and Alvarez-Buylla, 2019) during early life. In the adult brain, endogenous neurogenesis contributes significantly to neuroplasticity (Moreno-Jiménez et al., 2019) and neural repair following injury (Jessberger and Gage, 2014; Denoth-Lippuner and Jessberger, 2021). Notably, the cellular constituents of the neurogenic niche microenvironment, including glial cells (Kyle et al., 2019), exert a significant influence on adult neurogenesis.

Astrocytes, derived from the neuroectoderm (Kang et al., 2013), differentiate from radial glial cells early in development (Santos et al., 2017) and neural progenitor cells at later stages (Nagao et al., 2016). Comprising a significant portion of the central nervous system (CNS) (Hasel and Liddelow, 2021), astrocytes provide essential metabolic support to neurons (Hélène et al., 2023), contribute to calcium homeostasis (Jones et al., 2018), and supply neurotrophic factors (Zhang et al., 2020). Astrocyte dysfunction has profound implications for CNS health and function (Yang et al., 2022).

Mitochondria, double-membrane-bound organelles (Suomalainen and Nunnari, 2024), are primarily known for generating ATP through oxidative phosphorylation, placing them at the core of nutrient sensing and metabolic regulation. Beyond energy production, mitochondria participate in diverse cellular processes, including metabolism, metabolite and ion transport, apoptosis regulation, inflammation, signal transduction, and mitochondrial DNA inheritance (Szabo and Szewczyk, 2023). Mitochondrial function is inextricably linked to cellular function and profoundly influences overall cellular health. Activated astrocytes are a common feature in various neurodegenerative diseases and CNS injuries. Astrocytic mitochondrial function is crucial for maintaining overall brain metabolism, synaptic transmission, and neuroprotection. Impaired astrocytic mitochondrial function can lead to energy deficits, calcium dysregulation, inflammation, and glutamate imbalance (Gollihue and Norris, 2020). Furthermore, studies have revealed age-related changes in the distribution of astrocytic mitochondria within the neurogenic SVZ. In young mice, astrocytic mitochondria are perinuclear, exhibiting a dense matrix rich in cristae. In contrast, aged mice display a more dispersed cytoplasmic distribution of mitochondria in astrocytes, characterized by a lighter matrix, fewer cristae, and dilated cristae morphology (Capilla-Gonzalez et al., 2014). The findings underscore the dynamic nature of astrocytic mitochondria and their potential vulnerability to age-related decline and dysfunction.

The role of astrocytic mitochondria in neurogenesis remains relatively underexplored. A comprehensive understanding of this role is crucial for elucidating the pathophysiological mechanisms underlying neurological disorders, particularly those characterized by impaired neuronal function. The following section will examine the relationship between neurogenesis and central nervous system disorders, focusing on Alzheimer’s disease and depression as illustrative examples.

## 2 Central nervous system diseases and adult neurogenesis

**Figure 1:**
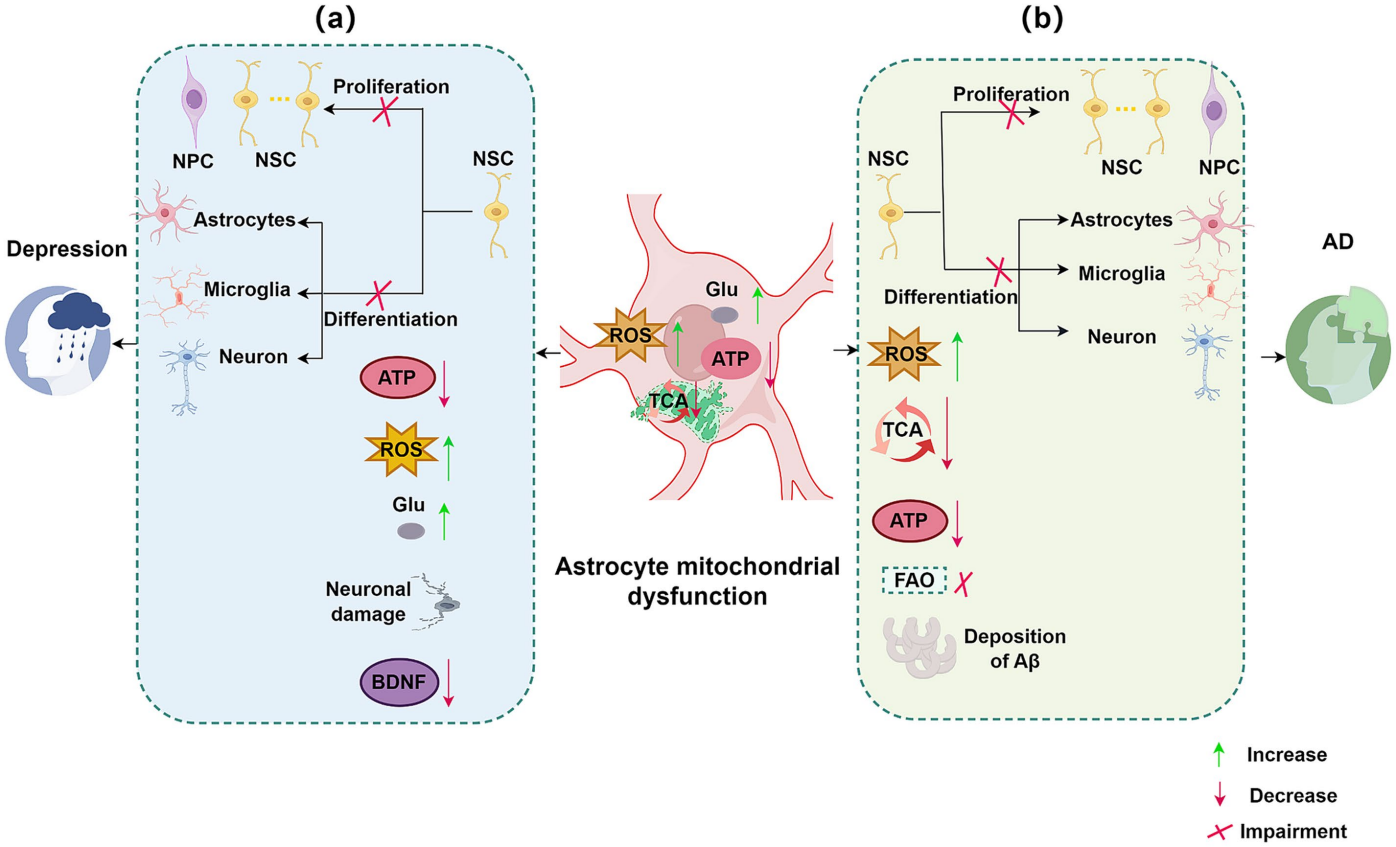
Impact of astrocytic mitochondrial dysfunction and impaired neurogenesis on neurological disorders. (a) Targeting astrocytic mitochondria and neurogenesis in depression. In depression, several signaling pathways within astrocytic mitochondria are impaired, including TCA signaling and glutamate uptake. Astrocytic mitochondrial dysfunction leads to decreased NSC proliferation and differentiation, reduced ATP and BDNF levels, increased ROS and Glu, and neuronal damage. (b) Targeting astrocytic mitochondria and neurogenesis in AD. In AD, astrocytic mitochondrial dysfunction affects pathways such as TCA cycle signaling and FAO. This dysfunction results in decreased NSC proliferation and differentiation, reduced ATP production, increased ROS, and accumulation of Aβ. NSC, neural stem cell; ROS, reactive oxygen species; Glu, glutamate; BDNF, brain-derived neurotrophic factor; TCA, tricarboxylic acid cycle; Aβ, amyloid-β plaques; AD, Alzheimer’s disease; FAO, fatty acid oxidation; ATP, adenosine triphosphate.

Alzheimer’s disease (AD) and major depressive disorder (MDD), two prevalent central nervous system disorders, are strongly associated with impaired neurogenesis. Astrocytic mitochondrial dysfunction can modulate neurogenesis, thereby influencing disease progression. This section focuses on these two disorders to elucidate the relationship between astrocytic mitochondrial dysfunction and the pathophysiology of AD and MDD, as illustrated in Figure 1.

### 2.1 Alzheimer’s disease

AD, the most prevalent neurodegenerative disorder, is characterized by progressive cognitive decline and currently lacks effective treatment. While amyloid-β (Aβ) and tau protein accumulation are central to AD pathogenesis (Sardar Sinha et al., 2018; Liang et al., 2021). A significant decline in neurogenesis is also a hallmark of AD pathophysiology, observed in both patients and animal models (Polis et al., 2020). This decline in neurogenesis, crucial for learning and memory, contributes significantly to the cognitive impairments seen in AD.

In AD, astrocytes, key players in supporting neurogenesis, exhibit a rapid response to injury, undergoing significant molecular, cellular, and morphological changes (Cassé et al., 2018). These alterations disrupt astrocytic support for neural stem cells and newborn neurons, impairing stem cell proliferation and progenitor migration.

Furthermore, astrocytic mitochondrial dysfunction, exacerbated by risk factors like the APOE4 genotype (Lin et al., 2019; Schmukler et al., 2020; Fortea et al., 2024), contributes directly to AD progression through oxidative stress, impaired fatty acid oxidation (FAO), glutamate accumulation, and energy deficits (Preman et al., 2021). This mitochondrial dysfunction further impairs neurogenesis, reduces neuronal plasticity, and exacerbates neuronal vulnerability to Aβ and tau pathologies, accelerating disease progression and ultimately contributing to the debilitating dementia characteristic of AD.

### 2.2 Major depressive disorder

MDD is a severe, chronic psychiatric illness with increasing prevalence and mortality, posing a substantial societal burden (Choi et al., 2019). Reduced neurogenesis in the dentate gyrus is considered a key factor in MDD pathogenesis (Jacobs et al., 2000). Astrocytes are crucial for maintaining neurogenesis, yet in preclinical models of depression, such as chronic mild stress (CMS), astrocytes exhibit mitochondrial dysfunction and reduced numbers (Shu et al., 2019). Moreover, compromising astrocytic function, for instance through lipopolysaccharide (LPS) exposure, induces mitochondrial damage and impairs neuronal synaptic plasticity (Li et al., 2023). Both mitochondrial uncoupling protein 2 (UCP2) and peroxisome proliferator-activated receptor gamma coactivator 1-alpha (PGC-1α) are linked to astrocytic mitochondrial function and implicated in MDD pathogenesis; UCP2 knockout mice display exacerbated depressive-like behaviors and impaired neurogenesis under CMS induction (Du et al., 2016). While PGC-1α deficiency in astrocytes disrupts astrocyte morphogenesis and neuronal synapse development (Zehnder et al., 2021).

This astrocytic mitochondrial dysfunction significantly contributes to the core clinical symptoms of MDD. The resulting reduction in neurogenesis, particularly within the hippocampus, contributes to depressed mood, anhedonia, and cognitive deficits. Furthermore, mitochondrial dysfunction leads to decreased ATP production and can contribute to extracellular glutamate (Glu) accumulation, both of which can exacerbate neuronal damage and dysfunction. Impaired astrocytic support, coupled with increased inflammation and oxidative stress, likely hinders the brain’s capacity to adapt to stress and form new positive associations, perpetuating the depressive state. The loss of astrocytic metabolic support further compromises neuronal function and resilience, exacerbating stress responses and potentially leading to anhedonia and lack of motivation. Moreover, disrupted neurotrophic factor signaling associated with astrocyte dysfunction can contribute to atrophy in brain regions associated with mood regulation, further compounding the severity of symptoms.

Given the crucial role of neurogenesis in both depression and Alzheimer’s disease, a deeper understanding of the mechanisms by which astrocytic mitochondria regulate this process is essential for elucidating the pathophysiology of these disorders and developing novel therapeutic strategies.

## 3 Role of astrocytic mitochondria in adult neurogenesis

To further explore how astrocytic mitochondria influence neurogenesis, we will delve into the specific mechanisms underlying their role in adult neurogenesis.

**Figure 2:**
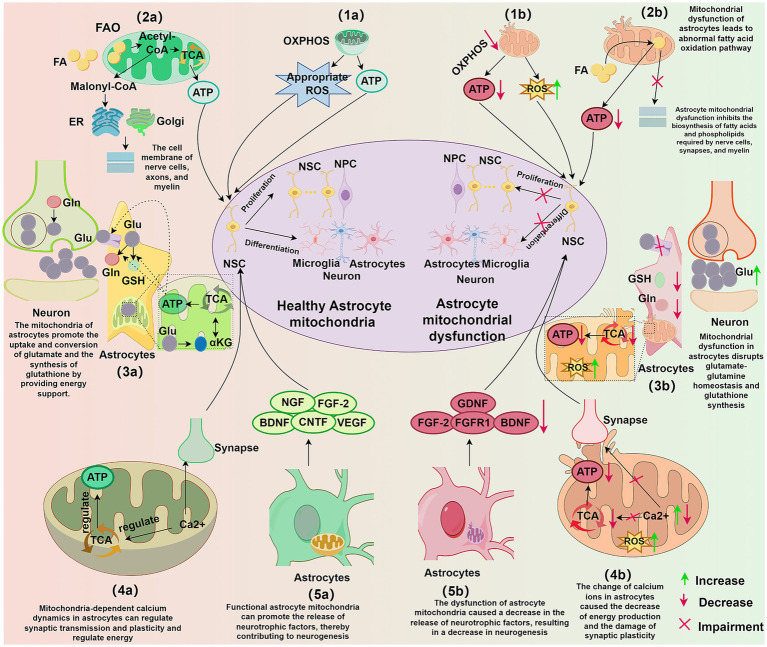
Influence of astrocytic mitochondria on neurogenesis. (1a) Functional astrocytic mitochondria through OXPHOS provide energy and appropriate levels of ROS for neurogenesis. (1b) Dysfunctional astrocytic mitochondria exhibit reduced OXPHOS, leading to decreased ATP production and increased ROS, impacting NSC proliferation and differentiation. (2a) FAO pathway in astrocytic mitochondria provides energy and synthesizes lipid substrates to support neuronal synapse formation, cell membrane synthesis, and myelination. (2b) Astrocytic mitochondrial dysfunction disrupts FAO, reducing ATP production and interfering with lipid homeostasis, inhibiting the synthesis of lipids required for neuronal synapses, cell membranes, and myelin. (3a) Functional astrocytic mitochondria provide energy for glutamine-glutamate homeostasis and GSH synthesis in astrocytes, supporting the development and maintenance of neural circuits. (3b) Astrocytic mitochondrial dysfunction disrupts glutamine-glutamate homeostasis and GSH synthesis, leading to neuronal excitotoxicity, decreased ATP, and increased ROS. (4a) Astrocytic mitochondria-dependent calcium dynamics regulate synaptic transmission and plasticity, modulate energy production, and directly influence the development and maintenance of neural circuits. (4b) Alterations in astrocytic calcium levels result in reduced energy production, increased ROS, and impaired synaptic plasticity. (5a) Functional astrocytic mitochondria promote the release of neurotrophic factors from astrocytes, thereby facilitating neurogenesis. (5b) Astrocytic mitochondrial dysfunction leads to decreased neurotrophic factor release, negatively impacting neurogenesis. OXPHOS, oxidative phosphorylation; ATP, adenosine triphosphate; ROS, reactive oxygen species; ATP, adenosine triphosphate; NSC, neural stem cell; NPC, neural progenitor cell; TCA, tricarboxylic acid cycle; ER, endoplasmic reticulum; Golgi, Golgi apparatus; FAO, fatty acid oxidation; FA, fatty acid; Glu, glutamate; Gln, glutamine; GSH, glutathione; αKG, α-ketoglutarate; Ca 2+, calcium ion; BDNF, brain-derived neurotrophic factor; NGF, nerve growth factor; FGF-2, fibroblast growth factor 2; CNTF, ciliary neurotrophic factor; VEGF, vascular endothelial growth factor; FGFR1, fibroblast growth factor receptor 1; GDNF, glial cell line-derived neurotrophic factor.

As depicted in Figure 2, astrocytic mitochondria influence neurogenesis through a variety of metabolic pathways, including oxidative phosphorylation, fatty acid oxidation, amino acid metabolism, and maintenance of calcium homeostasis. Furthermore, astrocytes support neurogenesis by secreting neurotrophic factors. The metabolic and regulatory functions act in concert to ensure proper neurogenic progression and contribute significantly to overall nervous system homeostasis.

### 3.1 Metabolic support

Astrocytes utilize diverse metabolic pathways, including glycolysis, the pentose phosphate pathway, and oxidative phosphorylation for glucose metabolism (Zhang et al., 2023). Fatty acid metabolism in astrocytes is characterized by fatty acid oxidation and sphingolipid metabolism, while amino acid metabolism involves neurotransmitter, serine, and kynurenine pathways. This metabolic support provides energy not only for astrocytes themselves but also for neurons, playing a critical role in maintaining neuronal health and facilitating interneuronal signaling. Neurogenesis requires extensive metabolic reprogramming (Maffezzini et al., 2020), including shifts in cellular energy sources. Mitochondria are central to this metabolic plasticity and are increasingly recognized as key regulators of neural stem cell fate and neurodevelopment (Khacho et al., 2019). Astrocytic mitochondria are heavily involved in oxidative phosphorylation, fatty acid oxidation, and amino acid metabolism, processes intimately linked to neurogenesis.

#### 3.1.1 Oxidative phosphorylation

Although astrocytes primarily rely on glycolysis for energy production (Vicente-Gutierrez et al., 2021), they can utilize oxidative phosphorylation within their mitochondria to rapidly generate ATP under conditions of high energy demand, sufficient oxygen supply, low lactate availability, or high lactate requirements. Neuronal differentiation of NSCs necessitates a metabolic shift toward oxidative phosphorylation (Bifari et al., 2020). This shift is not only crucial for meeting increased energy demands but also plays a significant role in regulating brain metabolism and antioxidant defense (Rose et al., 2020; Rubio-Atonal and Ioannou, 2023).

NSC proliferation and differentiation are energy-intensive processes (Cassiano et al., 2022). The metabolic switch in astrocytes from glycolysis to oxidative phosphorylation enhances energy supply, supporting these neurogenic processes (Chen et al., 2023). During oxidative phosphorylation, NADH and FADH2 serve as essential electron sources for the electron transport chain (ETC) (Melin and Hellwig, 2020), donating electrons to Complex I and Complex II, respectively, to drive ATP synthesis (Vasan et al., 2022). This process also generates ROS as a byproduct (Parousis et al., 2018). While excessive ROS can be detrimental to neuronal health (Sies et al., 2024), moderate ROS levels can induce neurogenesis (D’Angelo et al., 2018; Aravind et al., 2021).

ROS are proposed to play a key role in regulating stem cell homeostasis (Chen et al., 2021; Maraldi et al., 2021), influencing the reversible equilibrium between NSC quiescence and activation (Hwang et al., 2021). ROS modulate NSC fate through various signaling pathways. ROS activate the Nrf2-ARE pathway (Kahroba et al., 2021), promoting nuclear translocation of nuclear factor erythroid 2-related factor 2 (Nrf2). Nrf2 subsequently binds to the antioxidant response element (ARE), enhancing expression of antioxidant genes like heme oxygenase-1 (HO-1) and NAD(P)H quinone oxidoreductase 1 (NQO1) (Sun et al., 2021), thus protecting NSCs from oxidative damage. ROS also activate the PI3K/Akt pathway by phosphorylating and activating cell membrane growth factor receptors. Activated PI3K converts phosphatidylinositol 4,5-bisphosphate (PIP2) to phosphatidylinositol 3,4,5-trisphosphate (PIP3), recruiting and activating Akt (Le Belle et al., 2011; Hansen et al., 2019). Akt promotes NSC survival by inhibiting pro-apoptotic proteins (e.g., Bad) and upregulating anti-apoptotic proteins (e.g., Bcl-2) (Tao et al., 2017). Furthermore, ROS activate the MAPK/ERK pathway by stimulating cell surface receptors (e.g., growth factor receptors) or directly modulating intracellular signaling molecules, leading to RAS activation. This triggers a downstream cascade involving RAF kinase, MEK (MAPK/ERK kinase), and ultimately ERK phosphorylation (Zheng et al., 2021). Activated ERK translocates to the nucleus, regulating genes involved in cell proliferation and differentiation, such as Bcl-2 and Cyclin D1, promoting NSC differentiation (Kučera et al., 2016; Wang et al., 2021).

Under pathological conditions, inhibiting oxidative phosphorylation in astrocytes with mitochondrial inhibitors leads to significant ATP depletion (Astakhova et al., 2019), suggesting that reduced astrocytic oxidative phosphorylation can negatively impact NSC proliferation and differentiation by limiting energy availability. Studies have shown that NSCs accumulate ROS during reoxygenation, and inhibiting ROS biosynthesis counteracts their proliferation and neurogenic potential (Hameed et al., 2015). Conversely, excessive ROS can inhibit ETC Complex I activity (Dong et al., 2020) and drive stem cells out of quiescence, ultimately depleting the stem cell pool (Rossi et al., 2008). Furthermore, astrocyte-induced oxidative stress can contribute to neuronal death (Sulimai et al., 2021). These findings collectively demonstrate the multifaceted role of astrocytic oxidative phosphorylation in influencing neurogenesis through both ATP generation and ROS production.

#### 3.1.2 Fatty acid oxidation pathway

Astrocytic mitochondrial fatty acid oxidation (FAO) supports neurogenesis by providing both energy and metabolic intermediates (Lee et al., 2021). FAO involves the breakdown of fatty acids within the mitochondrial matrix into acetyl-CoA, releasing energy and generating substantial amounts of citrate, FADH2, and NADH to fuel oxidative phosphorylation (Mekala et al., 2021; Zhang T. et al., 2022), thereby providing energy for neurons (Lee et al., 2021). Citrate, an intermediate in the tricarboxylic acid (TCA) cycle, contributes to ATP production (Guo et al., 2022) and can also be converted to acetyl-CoA via ATP citrate lyase, influencing neurogenesis. Astrocytic FAO is particularly crucial for the synthesis of lipid membranes, especially those of neuronal cells (Ali and Szabó, 2023). Adult neural stem cells in the brain rely on FAO to support both aerobic respiration and proliferative activity (Stoll et al., 2015).

Acetyl-CoA is converted to malonyl-CoA, which is subsequently processed through the endoplasmic reticulum and Golgi apparatus to form palmitate, a key substrate for lipid synthesis (Wegner et al., 2021). Acetyl-CoA can induce neural stem cell exit from quiescence, enhance proliferation and differentiation (Zhou et al., 2019), and contribute to phospholipid synthesis, influencing neuronal synaptic strength, axonal growth, and cell membrane and myelin formation (Fadó et al., 2021; Roy and Tedeschi, 2021).

Studies have demonstrated that the brain heavily relies on astrocytic mitochondrial oxidative phosphorylation for fatty acid degradation and maintenance of lipid homeostasis. Astrocytic mitochondrial dysfunction can activate microglia and inhibit the biosynthesis of fatty acids and phospholipids required for myelin replenishment, gradually inducing neuroinflammation and neurodegeneration (Mi et al., 2023), thereby negatively impacting neurogenesis. Another study showed that knocking out carnitine palmitoyltransferase 1A (CPT1A), a key enzyme in mitochondrial FAO, in adult mouse astrocytes affects physiological ROS production (Morant-Ferrando et al., 2023), which can also influence neurogenesis. *In vitro* experiments have demonstrated that exposing quiescent adult neural stem cells to malonyl-CoA dose-dependently prevents quiescence induction and even promotes proliferation, indicating the importance of malonyl-CoA levels in regulating neural stem cell proliferation (Knobloch et al., 2017). These findings collectively highlight the influence of astrocytic mitochondrial FAO on neurogenesis through energy provision and the generation of key metabolic intermediates.

#### 3.1.3 Amino acid metabolic pathways

Astrocytic mitochondrial amino acid metabolism significantly influences neurogenesis (Guo et al., 2023), particularly through the synthesis and recycling of the neurotransmitter glutamate and the synthesis of antioxidants such as glutathione. A key astrocytic function is the rapid removal of neurotransmitters from the synaptic cleft (Dewa et al., 2024). In the glutamate-glutamine cycle, neurons and astrocytes cooperate closely through neurotransmitter recycling (Andersen et al., 2022). Glutamate-induced excitotoxicity is a recognized cause of neuronal cell death (Zhang Z. et al., 2022), and the uptake and conversion of glutamate is an energy-intensive process heavily reliant on mitochondrial energy supply (Havelund et al., 2019).

Astrocytes take up excess glutamate from the synaptic cleft via transporters to prevent excitotoxic neuronal damage (Qu et al., 2021). Within astrocytes, glutamate is converted to glutamine in the cytoplasm by glutamine synthetase (Luo et al., 2019). Alternatively, glutamate can be converted to *α*-ketoglutarate (αKG) within mitochondria through reactions catalyzed by glutamate dehydrogenase or aminotransferases (Pecchillo Cimmino et al., 2022). αKG, a key intermediate in the TCA cycle, plays a crucial role in cellular energy metabolism (Iwaniak et al., 2022). Glutamate is also a precursor for glutathione synthesis. In the cytoplasm, glutamate and cysteine are combined by cysteine ligase to form *γ*-glutamylcysteine. This dipeptide then reacts with glycine, in an ATP-dependent reaction catalyzed by glutathione synthetase, to form glutathione. Glutathione is a potent antioxidant crucial for maintaining mitochondrial integrity and function, scavenging free radicals and protecting mitochondria from oxidative damage. Astrocytic mitochondria, by providing energy, support glutamate uptake, conversion, and glutathione synthesis. This is essential for maintaining homeostasis in the glutamate-glutamine cycle and regulating the microenvironment necessary for neurogenesis. A balanced glutamate-glutamine cycle helps prevent excitotoxicity, while glutathione, through its antioxidant properties, mitigates oxidative stress-induced damage to neural cells, protecting neuronal function and neurogenesis.

Glutamate exhibits a biphasic dose–response on neurogenesis: low doses are beneficial, while excessive glutamate levels, due to increased release or decreased removal, can lead to neuronal atrophy and depression (Rubio-Casillas and Fernández-Guasti, 2016). Furthermore, modulating glutamate receptor subtypes can regulate NSC proliferation and differentiation. For instance, regulating metabotropic glutamate receptor subtype 4 (mGluR4) can influence NSC proliferation and apoptosis by inhibiting Cyclin D1 expression, promoting pro-caspase-8/9/3 activation, disrupting the balance between Bcl-2 and Bax (Bcl-2-associated X protein), and downregulating the expression of Gli-1, a transcription factor in the sonic hedgehog signaling pathway (Reichenbach et al., 2018; Zhang et al., 2018). Modulating metabotropic glutamate receptor subtype 5 (mGluR5) can influence neurogenesis by affecting the expression of the glutamate transporter SLC1A3 (also known as EAAT1/GLAST), thereby regulating stem cell activation and proliferation in different microenvironments. Astrocyte-derived mitochondrial ROS influence glucose utilization via glutathione metabolism, thereby modulating redox status and potentially neuronal survival (Vicente-Gutierrez et al., 2019). Glutathione depletion can exacerbate oxidative stress (Jaganjac et al., 2022), lead to mitochondrial dysfunction (Quinzii and Lopez, 2021), impair neural stem cell proliferation (Jeong et al., 2018) and differentiation (Aoyama, 2021), and increase apoptosis (Wang L. et al., 2020). In pathological conditions, astrocytic mitochondrial dysfunction disrupts glutamate metabolism and glutathione synthesis, contributing to neuronal damage and impaired neurogenesis through excitotoxicity and oxidative stress.

### 3.2 Calcium homeostasis

A key function of astrocytic mitochondria is buffering cytosolic calcium and regulating intracellular calcium homeostasis (Ahn et al., 2022; Lee et al., 2022), essential for cellular functions involved in neurogenesis. Calcium signaling networks profoundly influence neural stem cell proliferation, migration, and differentiation (Toth et al., 2016). Through mitochondrial regulation of calcium signaling, astrocytes exert multi-faceted control over neurogenesis (Glaser et al., 2019).

Firstly, astrocytes, via mitochondrial calcium signaling, influence energy metabolism and modulate synaptic activity. For example, mitochondrial calcium status regulates astrocytic glutamate uptake and vesicular release, impacting neuronal energy supply and synaptic transmission strength (Hirrlinger and Nimmerjahn, 2022; Satarker et al., 2022). Mitochondrial calcium also modulates the activity of TCA cycle enzymes, influencing ATP production (Assis et al., 2022). Furthermore, the action of mitochondrial proteins determines mitochondrial localization within astrocytes, impacting calcium wave propagation, mitochondrial energy production, and the regulation of neuronal function (Stephen et al., 2015). Critically, mitochondria-dependent calcium dynamics in astrocytes regulate synaptic transmission and plasticity (Serrat et al., 2021), directly influencing the development and maintenance of neural circuits. This mitochondria-based calcium signaling ensures adequate energy supply for astrocytes and provides essential metabolic support and signaling cues to neural stem cells, ultimately impacting neurogenesis.

Recent studies have shown that autoimmune inflammation can disrupt astrocytic calcium signaling, leading to increased glutamatergic gliotransmission and impaired astrocyte-mediated synaptic plasticity (Baraibar et al., 2024). This calcium dysregulation disrupts neuron-astrocyte communication and weakens synaptic stability and function, negatively impacting neuronal health. Mitochondria, by regulating intracellular calcium levels, play a critical role in maintaining cell survival, indirectly influencing neurogenesis (Klocke et al., 2023). Excessive calcium accumulation can trigger opening of the mitochondrial permeability transition pore (mPTP), leading to astrocyte death (Sun et al., 2018; Bauer and Murphy, 2020). This cell death compromises astrocytic support for surrounding neurons, impairing neuronal health and disrupting neurogenesis. Mitochondria-regulated calcium signaling can also activate specific signaling pathways, such as NF-κB or CREB, which regulate gene expression related to neurogenesis, directly impacting neural stem cell proliferation and differentiation. Through the modulation of these transcription factors, calcium signaling plays a significant role in the pathological regulation of neurogenesis (Huang et al., 2022).

In summary, by maintaining calcium homeostasis, astrocytic mitochondria exert profound effects on synaptic activity, synaptic plasticity, and cell survival, particularly under pathological conditions, thereby influencing the progression of neurogenesis.

### 3.3 Neurotrophic factor secretion

Astrocytic mitochondria influence the production and release of various neurotrophic factors (Ren et al., 2020; Zhao et al., 2021), which play a critical role in neurogenesis (Preez et al., 2021). Studies have demonstrated that neurotrophic factors secreted by astrocytes, such as brain-derived neurotrophic factor (BDNF), nerve growth factor (NGF), and fibroblast growth factor 2 (FGF2), promote neuroblast migration, neural stem cell proliferation, survival, and differentiation (Brown et al., 2021; Lin et al., 2021; Wu et al., 2023). Astrocyte-derived ciliary neurotrophic factor (CNTF) enhances neurogenesis by promoting NSC proliferation and differentiation via the FAK-JNK pathway and by modulating the expression of related cytokines such as leukemia inhibitory factor (LIF) and interleukin-6 (IL-6) (Jia et al., 2018). Vascular endothelial growth factor (VEGF) also stimulates neurogenesis by binding to its receptor and activating multiple signaling pathways, including PI3K/Akt and MAPK (Preez et al., 2021; van den Berg et al., 2021).

Under pathological conditions, downregulation of astrocytic FGF2 and its receptor, FGFR1, impairs glutamatergic synapse formation and ultimately reduces neurogenesis (Choi et al., 2022). Studies of astrocytes under recurrent hypoglycemic conditions, both *in vivo* and *in vitro*, have revealed mitochondrial dysfunction and decreased secretion of BDNF and glial cell line-derived neurotrophic factor (GDNF). Protecting mitochondrial function restores astrocyte viability and neurotrophic factor production and secretion (Gao et al., 2021). These findings collectively demonstrate that astrocytic mitochondria influence various stages of neurogenesis through the modulation of neurotrophic factor production and release.

## 4 Therapeutic strategy for neurological disorders

The previous chapter detailed the mechanisms by which astrocytic mitochondria influence adult neurogenesis. Building upon these findings, this chapter will explore potential therapeutic strategies targeting astrocytic mitochondria to promote neurogenesis and ameliorate neurological disease symptoms, focusing on Alzheimer’s disease and depression as illustrative examples.

Targeting astrocytic dysfunction, particularly mitochondrial impairment, represents a promising therapeutic strategy for AD (Galea et al., 2022). Restoring astrocytic energy metabolism has demonstrated the potential to partially reverse AD pathology and ameliorate clinical symptoms (Mamelak, 2017). Specifically, addressing APOE4-induced mitochondrial dysfunction and autophagy deficits offers compelling targets for pharmacological intervention. For example *in vitro* studies reveal that APOE4-expressing astrocytes negatively impact dendritic spine dynamics in neuron-astrocyte co-cultures (Lee et al., 2023), suggesting that early intervention targeting APOE4-mediated mitochondrial dysfunction may be crucial for delaying AD progression. These findings underscore the profound impact of astrocytic mitochondrial dysfunction on AD pathophysiology and highlight the therapeutic potential of targeting these mechanisms.

In the context of depression, therapeutic strategies targeting astrocytic mitochondria and neurogenesis are emerging. Mitochondrial transplantation has shown promise in preclinical models, ameliorating LPS-induced depressive-like behavior by reducing neuroinflammation and increasing BDNF expression and neurogenesis (Wang et al., 2019). Modulating astrocytic sigma-1 receptor (Sig-1R) activity promotes mitochondrial transfer from astrocytes to neurons, enhancing neuronal survival and exerting antidepressant-like effects (Wang Y. et al., 2020). Furthermore, targeting the RARγ-GLT-1 pathway in astrocytes within retinoic acid-induced depression models promotes neurogenesis and mitigates depressive-like behavior (Huang et al., 2024). These findings emphasize the therapeutic potential of targeting astrocytic mitochondria to enhance neurogenesis and alleviate depressive symptoms.

## 5 Summary

Endogenous neurogenesis, with its inherent self-repair capabilities, potential for long-term therapeutic effects, and relatively low risk of side effects, represents a promising therapeutic strategy for neurological disorders. Enhancing endogenous neurogenesis has become a focal point of current research, with mitochondria playing a crucial role in this process. Current drug development efforts predominantly focus on directly modulating neuronal function, often overlooking the contribution of astrocytes. Future research should prioritize investigating the reactive changes in astrocytes during disease progression and their direct impact on neurogenesis. Specifically, exploring astrocytic mitophagy, mitochondrial dynamics, biogenesis, and energy metabolism will likely reveal detailed mechanisms underlying their influence on neurogenesis. This knowledge promises to uncover novel therapeutic avenues for neurological disorders, deepen our understanding of neural stem cell regulation, and ultimately lead to the development of more effective treatments.

## References

[ref1] Ahn B. Ranjit R. Kneis P. Xu H. Piekarz K. M. Freeman W. M.. (2022). Scavenging mitochondrial hydrogen peroxide by peroxiredoxin 3 overexpression attenuates contractile dysfunction and muscle atrophy in a murine model of accelerated sarcopenia. Aging Cell 21:e13569. doi: 10.1111/acel.13569, PMID: 35199907 PMC8920438

[ref2] Ali O. Szabó A. (2023). Review of eukaryote cellular membrane lipid composition, with special attention to the fatty acids. Int. J. Mol. Sci. 24:15693. doi: 10.3390/ijms242115693 37958678 PMC10649022

[ref3] Allen N. J. Barres B. A. (2009). Neuroscience: glia - more than just brain glue. Nature 457, 675–677. doi: 10.1038/457675a, PMID: 19194443

[ref4] Altman J. (1962). Are new neurons formed in the brains of adult mammals? Science 135, 1127–1128. doi: 10.1126/science.135.3509.1127 13860748

[ref5] Altman J. Das G. D. (1965). Autoradiographic and histological evidence of postnatal hippocampal neurogenesis in rats. J. Comp. Neurol. 124, 319–335. doi: 10.1002/cne.901240303 5861717

[ref6] Alvarez-Buylla A. Garcia-Verdugo J. M. (2002). Neurogenesis in adult subventricular zone. J. Neurosci. 22, 629–634. doi: 10.1523/JNEUROSCI.22-03-00629.2002, PMID: 11826091 PMC6758521

[ref7] Andersen J. V. Schousboe A. Verkhratsky A. (2022). Astrocyte energy and neurotransmitter metabolism in Alzheimer's disease: integration of the glutamate/GABA-glutamine cycle. Prog. Neurobiol. 217:102331. doi: 10.1016/j.pneurobio.2022.102331, PMID: 35872221

[ref8] Aoyama K. (2021). Glutathione in the brain. Int. J. Mol. Sci. 22:5010. doi: 10.3390/ijms22095010, PMID: 34065042 PMC8125908

[ref9] Aravind P. Bulbule S. R. Hemalatha N. Babu R. L. Devaraju K. S. (2021). Elevation of gene expression of calcineurin, calmodulin and calsyntenin in oxidative stress induced PC12 cells. Genes Dis. 8, 87–93. doi: 10.1016/j.gendis.2019.09.001, PMID: 33569517 PMC7859428

[ref10] Assis L. H. P. Dorighello G. G. Rentz T. de Souza J. C. Vercesi A. E. de Oliveira H. C. F. (2022). *In vivo* pravastatin treatment reverses hypercholesterolemia induced mitochondria-associated membranes contact sites, foam cell formation, and phagocytosis in macrophages. Front. Mol. Biosci. 9:839428. doi: 10.3389/fmolb.2022.839428, PMID: 35372506 PMC8965079

[ref11] Astakhova A. Chistyakov D. Thomas D. Geisslinger G. Brüne B. Sergeeva M.. (2019). Inhibitors of oxidative phosphorylation modulate astrocyte inflammatory responses through AMPK-dependent Ptgs2 mRNA stabilization. Cells 8:1185. doi: 10.3390/cells8101185, PMID: 31581537 PMC6829456

[ref12] Baraibar A. M. Colomer T. Moreno-García A. Bernal-Chico A. Sánchez-Martín E. Utrilla C.. (2024). Autoimmune inflammation triggers aberrant astrocytic calcium signaling to impair synaptic plasticity. Brain Behav. Immun. 121, 192–210. doi: 10.1016/j.bbi.2024.07.010, PMID: 39032542 PMC11415231

[ref13] Bauer T. M. Murphy E. (2020). Role of mitochondrial calcium and the permeability transition pore in regulating cell death. Circ. Res. 126, 280–293. doi: 10.1161/CIRCRESAHA.119.316306, PMID: 31944918 PMC8317591

[ref14] Bifari F. Dolci S. Bottani E. Pino A. Di Chio M. Zorzin S.. (2020). Complete neural stem cell (NSC) neuronal differentiation requires a branched chain amino acids-induced persistent metabolic shift towards energy metabolism. Pharmacol. Res. 158:104863. doi: 10.1016/j.phrs.2020.104863, PMID: 32407957

[ref15] Bosworth A. P. Allen N. J. (2017). The diverse actions of astrocytes during synaptic development. Curr. Opin. Neurobiol. 47, 38–43. doi: 10.1016/j.conb.2017.08.017, PMID: 28938161

[ref16] Bradl M. Lassmann H. (2010). Oligodendrocytes: biology and pathology. Acta Neuropathol. 119, 37–53. doi: 10.1007/s00401-009-0601-5, PMID: 19847447 PMC2799635

[ref17] Brown C. McKee C. Halassy S. Kojan S. Feinstein D. L. Chaudhry G. R. (2021). Neural stem cells derived from primitive mesenchymal stem cells reversed disease symptoms and promoted neurogenesis in an experimental autoimmune encephalomyelitis mouse model of multiple sclerosis. Stem Cell Res Ther 12:499. doi: 10.1186/s13287-021-02563-8, PMID: 34503569 PMC8427882

[ref18] Capilla-Gonzalez V. Cebrian-Silla A. Guerrero-Cazares H. Garcia-Verdugo J. M. Quiñones-Hinojosa A. (2014). Age-related changes in astrocytic and ependymal cells of the subventricular zone. Glia 62, 790–803. doi: 10.1002/glia.22642 24677590 PMC4322944

[ref19] Cassé F. Richetin K. Toni N. (2018). Astrocytes’ contribution to adult neurogenesis in physiology and Alzheimer’s disease. Front. Cell. Neurosci. 12:432. doi: 10.3389/fncel.2018.00432, PMID: 30538622 PMC6277517

[ref20] Cassiano L. M. G. Oliveira M. S. Pioline J. Salim A. C. M. Coimbra R. S. (2022). Neuroinflammation regulates the balance between hippocampal neuron death and neurogenesis in an *ex vivo* model of thiamine deficiency. J. Neuroinflammation 19:272. doi: 10.1186/s12974-022-02624-6, PMID: 36376954 PMC9664832

[ref21] Chen F. Su R. Ni S. Liu Y. Huang J. Li G.. (2021). Context-dependent responses of Drosophila intestinal stem cells to intracellular reactive oxygen species. Redox Biol. 39:101835. doi: 10.1016/j.redox.2020.101835, PMID: 33360688 PMC7772796

[ref22] Chen Z. Yuan Z. Yang S. Zhu Y. Xue M. Zhang J.. (2023). Brain energy metabolism: astrocytes in neurodegenerative diseases. CNS Neurosci. Ther. 29, 24–36. doi: 10.1111/cns.13982, PMID: 36193573 PMC9804080

[ref23] Choi G. E. Chae C. W. Park M. R. Yoon J. H. Jung Y. H. Lee H. J.. (2022). Prenatal glucocorticoid exposure selectively impairs neuroligin 1-dependent neurogenesis by suppressing astrocytic FGF2-neuronal FGFR1 axis. Cell. Mol. Life Sci. 79:294. doi: 10.1007/s00018-022-04313-2, PMID: 35562616 PMC9106608

[ref24] Choi K. W. Chen C. Y. Stein M. B. Klimentidis Y. C. Wang M. J. Koenen K. C.. (2019). Assessment of bidirectional relationships between physical activity and depression among adults: a 2-sample Mendelian randomization study. JAMA Psychiatry 76, 399–408. doi: 10.1001/jamapsychiatry.2018.4175, PMID: 30673066 PMC6450288

[ref25] D’Angelo M. Antonosante A. Castelli V. Catanesi M. Moorthy N. Iannotta D.. (2018). PPARs and energy metabolism adaptation during neurogenesis and neuronal maturation. Int. J. Mol. Sci. 19:1869. doi: 10.3390/ijms19071869, PMID: 29949869 PMC6073366

[ref26] Dario B. Werner D. Stephanie L. Annika Z. Alessandro P. (2021). Mitochondria in neurogenesis: implications for mitochondrial diseases. Stem Cells 39, 1289–1297. doi: 10.1002/stem.3425 34089537

[ref27] Denoth-Lippuner A. Jessberger S. (2021). Formation and integration of new neurons in the adult hippocampus. Nat. Rev. Neurosci. 22, 223–236. doi: 10.1038/s41583-021-00433-z 33633402

[ref28] Dewa K. I. Arimura N. Kakegawa W. Itoh M. Adachi T. Miyashita S.. (2024). Neuronal DSCAM regulates the peri-synaptic localization of GLAST in Bergmann glia for functional synapse formation. Nat. Commun. 15:458. doi: 10.1038/s41467-023-44579-z, PMID: 38302444 PMC10834496

[ref29] Dong W. Luo B. Qiu C. Jiang X. Shen B. Zhang L.. (2020). TRIM3 attenuates apoptosis in Parkinson's disease via activating PI3K/AKT signal pathway. Aging (Albany NY) 13, 735–749. doi: 10.18632/aging.202181, PMID: 33253119 PMC7835008

[ref30] Du R. H. Wu F. F. Lu M. Shu X. D. Ding J. H. Wu G.. (2016). Uncoupling protein 2 modulation of the NLRP3 inflammasome in astrocytes and its implications in depression. Redox Biol. 9, 178–187. doi: 10.1016/j.redox.2016.08.006, PMID: 27566281 PMC5007434

[ref31] Du F. Yu Q. Chen A. Chen D. Yan S. S. (2018). Astrocytes attenuate mitochondrial dysfunctions in human dopaminergic neurons derived from iPSC. Stem Cell Rep. 10, 366–374. doi: 10.1016/j.stemcr.2017.12.021, PMID: 29396183 PMC5830955

[ref32] Fadó R. Rodríguez-Rodríguez R. Casals N. (2021). The return of malonyl-CoA to the brain: cognition and other stories. Prog. Lipid Res. 81:101071. doi: 10.1016/j.plipres.2020.101071, PMID: 33186641

[ref33] Fortea J. Pegueroles J. Alcolea D. Belbin O. Dols-Icardo O. Vaqué-Alcázar L.. (2024). APOE4 homozygosity represents a distinct genetic form of Alzheimer’s disease. Nat. Med. 30, 1284–1291. doi: 10.1038/s41591-024-02931-w, PMID: 38710950 PMC13310155

[ref34] Gage F. H. (2000). Mammalian neural stem cells. Science 287, 1433–1438. doi: 10.1126/science.287.5457.1433 10688783

[ref35] Gage F. H. (2019). Adult neurogenesis in mammals. Science 364, 827–828. doi: 10.1126/science.aav6885 31147506

[ref36] Galea E. Weinstock L. D. Larramona-Arcas R. Pybus A. F. Giménez-Llort L. Escartin C.. (2022). Multi-transcriptomic analysis points to early organelle dysfunction in human astrocytes in Alzheimer's disease. Neurobiol. Dis. 166:105655. doi: 10.1016/j.nbd.2022.105655, PMID: 35143967 PMC9504227

[ref37] Gao R. Ren L. Zhou Y. Wang L. Xie Y. Zhang M.. (2021). Recurrent non-severe hypoglycemia aggravates cognitive decline in diabetes and induces mitochondrial dysfunction in cultured astrocytes. Mol. Cell. Endocrinol. 526:111192. doi: 10.1016/j.mce.2021.111192, PMID: 33545179

[ref38] Glaser T. Arnaud Sampaio V. F. Lameu C. Ulrich H. (2019). Calcium signalling: A common target in neurological disorders and neurogenesis. Semin. Cell Dev. Biol. 95, 25–33. doi: 10.1016/j.semcdb.2018.12.002, PMID: 30529426

[ref39] Gollihue J. L. Norris C. M. (2020). Astrocyte mitochondria: central players and potential therapeutic targets for neurodegenerative diseases and injury. Ageing Res. Rev. 59:101039. doi: 10.1016/j.arr.2020.101039, PMID: 32105849 PMC7422487

[ref40] Guo D. He H. Meng Y. Luo S. Lu Z. (2022). Determiners of cell fates: the tricarboxylic acid cycle versus the citrate-malate shuttle. Protein Cell 14, 162–164. doi: 10.1093/procel/pwac026 PMC10098035 37051670

[ref41] Guo Y. Luo X. Guo W. (2023). The impact of amino acid metabolism on adult neurogenesis. Biochem. Soc. Trans. 51, 233–244. doi: 10.1042/BST20220762, PMID: 36606681

[ref42] Hameed L. S. Berg D. A. Belnoue L. Jensen L. D. Cao Y. Simon A. (2015). Environmental changes in oxygen tension reveal ROS-dependent neurogenesis and regeneration in the adult newt brain. eLife 4:e08422. doi: 10.7554/eLife.08422, PMID: 26485032 PMC4635398

[ref43] Hansen T. Thant C. White J. A. 2nd Banerjee R. Thuamsang B. Gunawardena S. (2019). Excess active P13K rescues huntingtin-mediated neuronal cell death but has no effect on axonal transport defects. Apoptosis 24, 341–358. doi: 10.1007/s10495-019-01520-4, PMID: 30725352 PMC6486460

[ref44] Hasel P. Liddelow S. A. (2021). Astrocytes. Curr. Biol. 31, R326–r327. doi: 10.1016/j.cub.2021.01.056 33848482

[ref45] Havelund J. F. Nygaard K. H. Nielsen T. H. Nordström C. H. Poulsen F. R. Færgeman N. J.. (2019). *In vivo* microdialysis of endogenous and (13)C-labeled TCA metabolites in rat brain: reversible and persistent effects of mitochondrial inhibition and transient cerebral ischemia. Meta 9:204. doi: 10.3390/metabo9100204, PMID: 31569792 PMC6835622

[ref46] Hélène R. Luc P. Anne-Karine B. S. (2023). Astrocytes as metabolic suppliers to support neuronal activity and brain functions. Essays Biochem. 67, 27–37. doi: 10.1042/EBC20220080 36504117 PMC10011397

[ref47] Hirrlinger J. Nimmerjahn A. (2022). A perspective on astrocyte regulation of neural circuit function and animal behavior. Glia 70, 1554–1580. doi: 10.1002/glia.24168, PMID: 35297525 PMC9291267

[ref48] Huang H. Lu W. Luo R. Zeng Y. Zhang Y. Su X.. (2024). Astrocytic RARγ mediates hippocampal astrocytosis and neurogenesis deficits in chronic retinoic acid-induced depression. Neuropsychopharmacology 3:1983. doi: 10.1038/s41386-024-01983-3 PMC11632084 39242924

[ref49] Huang J. U K. P. Yang F. Ji Z. Lin J. Weng Z.. (2022). Human pluripotent stem cell-derived ectomesenchymal stromal cells promote more robust functional recovery than umbilical cord-derived mesenchymal stromal cells after hypoxic-ischaemic brain damage. Theranostics 12, 143–166. doi: 10.7150/thno.57234, PMID: 34987639 PMC8690936

[ref50] Hwang I. Tang D. Paik J. (2021). Oxidative stress sensing and response in neural stem cell fate. Free Radic. Biol. Med. 169, 74–83. doi: 10.1016/j.freeradbiomed.2021.03.043, PMID: 33862161 PMC9594080

[ref51] Iwaniak P. Tomaszewska E. Muszyński S. Marszałek-Grabska M. Pierzynowski S. G. Dobrowolski P. (2022). Dietary alpha-Ketoglutarate partially abolishes adverse changes in the small intestine after gastric bypass surgery in a rat model. Nutrients 14:2062. doi: 10.3390/nu14102062, PMID: 35631203 PMC9146360

[ref52] Iwata R. Casimir P. Vanderhaeghen P. (2020). Mitochondrial dynamics in postmitotic cells regulate neurogenesis. Science (New York, N.Y.) 369, 858–862. doi: 10.1126/science.aba9760, PMID: 32792401

[ref53] Jacobs B. L. van Praag H. Gage F. H. (2000). Adult brain neurogenesis and psychiatry: a novel theory of depression. Mol. Psychiatry 5, 262–269. doi: 10.1038/sj.mp.4000712, PMID: 10889528

[ref54] Jaganjac M. Milkovic L. Zarkovic N. Zarkovic K. (2022). Oxidative stress and regeneration. Free Radic. Biol. Med. 181, 154–165. doi: 10.1016/j.freeradbiomed.2022.02.004 35149216

[ref55] Jeong E. M. Yoon J.-H. Lim J. Shin J.-W. Cho A. Y. Heo J.. (2018). Real-time monitoring of glutathione in living cells reveals that high glutathione levels are required to maintain stem cell function. Stem Cell Rep. 10, 600–614. doi: 10.1016/j.stemcr.2017.12.007, PMID: 29307581 PMC5830891

[ref56] Jessberger S. Gage F. H. (2014). Adult neurogenesis: bridging the gap between mice and humans. Trends Cell Biol. 24, 558–563. doi: 10.1016/j.tcb.2014.07.003 25124338

[ref57] Jia C. Keasey M. P. Lovins C. Hagg T. (2018). Inhibition of astrocyte FAK-JNK signaling promotes subventricular zone neurogenesis through CNTF. Glia 66, 2456–2469. doi: 10.1002/glia.23498, PMID: 30500112 PMC6863602

[ref58] Jones J. R. Kong L. Hanna M. G. IV Hoffman B. Krencik R. Bradley R.. (2018). Mutations in GFAP disrupt the distribution and function of organelles in human astrocytes. Cell Rep. 25, 947–958.e4. doi: 10.1016/j.celrep.2018.09.083, PMID: 30355500 PMC6275075

[ref59] Kahroba H. Ramezani B. Maadi H. Sadeghi M. R. Jaberie H. Ramezani F. (2021). The role of Nrf2 in neural stem/progenitors cells: from maintaining stemness and self-renewal to promoting differentiation capability and facilitating therapeutic application in neurodegenerative disease. Ageing Res. Rev. 65:101211. doi: 10.1016/j.arr.2020.101211, PMID: 33186670

[ref60] Kang Z. Wang C. Zepp J. Wu L. Sun K. Zhao J.. (2013). Act1 mediates IL-17-induced EAE pathogenesis selectively in NG2+ glial cells. Nat. Neurosci. 16, 1401–1408. doi: 10.1038/nn.3505, PMID: 23995070 PMC4106025

[ref61] Kaplan M. S. Hinds J. W. (1977). Neurogenesis in the adult rat: electron microscopic analysis of light radioautographs. Science 197, 1092–1094. doi: 10.1126/science.887941, PMID: 887941

[ref62] Kempermann G. (2012). New neurons for 'survival of the fittest'. Nat. Rev. Neurosci. 13, 727–736. doi: 10.1038/nrn3319 22948073

[ref63] Khacho M. Clark A. Svoboda D. S. MacLaurin J. G. Lagace D. C. Park D. S.. (2017). Mitochondrial dysfunction underlies cognitive defects as a result of neural stem cell depletion and impaired neurogenesis. Hum. Mol. Genet. 26, 3327–3341. doi: 10.1093/hmg/ddx217, PMID: 28595361 PMC5886206

[ref64] Khacho M. Harris R. Slack R. S. (2019). Mitochondria as central regulators of neural stem cell fate and cognitive function. Nat. Rev. Neurosci. 20, 34–48. doi: 10.1038/s41583-018-0091-3, PMID: 30464208

[ref65] Klocke B. Krone K. Tornes J. Moore C. Ott H. Pitychoutis P. M. (2023). Insights into the role of intracellular calcium signaling in the neurobiology of neurodevelopmental disorders. Front. Neurosci. 17:1093099. doi: 10.3389/fnins.2023.1093099 36875674 PMC9975342

[ref66] Knobloch M. Pilz G.-A. Ghesquière B. Kovacs W. J. Wegleiter T. Moore D. L.. (2017). A fatty acid oxidation-dependent metabolic shift regulates adult neural stem cell activity. Cell Rep. 20, 2144–2155. doi: 10.1016/j.celrep.2017.08.029, PMID: 28854364 PMC5583518

[ref67] Kochan S. M. V. Malo M. C. Jevtic M. Jahn-Kelleter H. M. Wani G. A. Ndoci K.. (2024). Enhanced mitochondrial fusion during a critical period of synaptic plasticity in adult-born neurons. Neuron 112, 1997–2014.e6. doi: 10.1016/j.neuron.2024.03.013, PMID: 38582081

[ref68] Kostović I. Sedmak G. Judaš M. (2019). Neural histology and neurogenesis of the human fetal and infant brain. NeuroImage 188, 743–773. doi: 10.1016/j.neuroimage.2018.12.043 30594683

[ref69] Kučera J. Binó L. Štefková K. Jaroš J. Vašíček O. Večeřa J.. (2016). Apocynin and Diphenyleneiodonium induce oxidative stress and modulate PI3K/Akt and MAPK/Erk activity in mouse embryonic stem cells. Oxidative Med. Cell. Longev. 2016:7409196. doi: 10.1155/2016/7409196, PMID: 26788250 PMC4691611

[ref70] Kuramoto Y. Fujita M. Takagi T. Takeda Y. Doe N. Yamahara K.. (2022). Early-phase administration of human amnion-derived stem cells ameliorates neurobehavioral deficits of intracerebral hemorrhage by suppressing local inflammation and apoptosis. J. Neuroinflammation 19:48. doi: 10.1186/s12974-022-02411-3, PMID: 35151317 PMC8840774

[ref71] Kyle J. Wu M. Gourzi S. Tsirka S. E. (2019). Proliferation and differentiation in the adult subventricular zone are not affected by CSF1R inhibition. Front. Cell. Neurosci. 13:97. doi: 10.3389/fncel.2019.00097, PMID: 31001085 PMC6454047

[ref72] Le Belle J. E. Orozco N. M. Paucar A. A. Saxe J. P. Mottahedeh J. Pyle A. D.. (2011). Proliferative neural stem cells have high endogenous ROS levels that regulate self-renewal and neurogenesis in a PI3K/Akt-dependant manner. Cell Stem Cell 8, 59–71. doi: 10.1016/j.stem.2010.11.028, PMID: 21211782 PMC3018289

[ref73] Lee H. Cho S. Kim M.-J. Park Y. J. Cho E. Jo Y. S.. (2023). ApoE4-dependent lysosomal cholesterol accumulation impairs mitochondrial homeostasis and oxidative phosphorylation in human astrocytes. Cell Rep. 42:113183. doi: 10.1016/j.celrep.2023.113183, PMID: 37777962

[ref74] Lee J. A. K. Hall B. Allsop J. Alqarni R. Allen S. P. (2021). Lipid metabolism in astrocytic structure and function. Semin. Cell Dev. Biol. 112, 123–136. doi: 10.1016/j.semcdb.2020.07.017 32773177

[ref75] Lee H. G. Wheeler M. A. Quintana F. J. (2022). Function and therapeutic value of astrocytes in neurological diseases. Nat. Rev. Drug Discov. 21, 339–358. doi: 10.1038/s41573-022-00390-x, PMID: 35173313 PMC9081171

[ref76] Li Y. Li J. Yang L. Ren F. Dong K. Zhao Z.. (2023). Ginsenoside Rb1 protects hippocampal neurons in depressed rats based on mitophagy-regulated astrocytic pyroptosis. Phytomedicine 121:155083. doi: 10.1016/j.phymed.2023.155083, PMID: 37722244

[ref77] Liang C. Zou T. Zhang M. Fan W. Zhang T. Jiang Y.. (2021). MicroRNA-146a switches microglial phenotypes to resist the pathological processes and cognitive degradation of Alzheimer's disease. Theranostics 11, 4103–4121. doi: 10.7150/thno.53418, PMID: 33754051 PMC7977456

[ref78] Lin M. S. Chiu I. H. Lin C. C. (2021). Ultrarapid inflammation of the olfactory bulb after spinal cord injury: protective effects of the granulocyte Colony-stimulating factor on early neurodegeneration in the brain. Front. Aging Neurosci. 13:701702. doi: 10.3389/fnagi.2021.701702, PMID: 34248610 PMC8267925

[ref79] Lin Z. Sur S. Soldan A. Pettigrew C. Miller M. Oishi K.. (2019). Brain oxygen extraction by using MRI in older individuals: relationship to Apolipoprotein E genotype and amyloid burden. Radiology 292, 140–148. doi: 10.1148/radiol.2019182726, PMID: 31012816 PMC6604795

[ref80] Luo L. L. Li Y. F. Shan H. M. Wang L. P. Yuan F. Ma Y. Y.. (2019). L-glutamine protects mouse brain from ischemic injury via up-regulating heat shock protein 70. CNS Neurosci. Ther. 25, 1030–1041. doi: 10.1111/cns.13184, PMID: 31218845 PMC6698979

[ref81] Maffezzini C. Calvo-Garrido J. Wredenberg A. Freyer C. (2020). Metabolic regulation of neurodifferentiation in the adult brain. Cell. Mol. Life Sci. 77, 2483–2496. doi: 10.1007/s00018-019-03430-9, PMID: 31912194 PMC7320050

[ref82] Mamelak M. (2017). Energy and the Alzheimer brain. Neurosci. Biobehav. Rev. 75, 297–313. doi: 10.1016/j.neubiorev.2017.02.001 28193453

[ref83] Maraldi T. Angeloni C. Prata C. Hrelia S. (2021). NADPH oxidases: redox regulators of stem cell fate and function. Antioxidants 10:973. doi: 10.3390/antiox10060973, PMID: 34204425 PMC8234808

[ref84] Mekala N. Kurdys J. Vicenzi A. P. Weiler L. R. Avramut C. Vazquez E. J.. (2021). MiR 208a regulates mitochondrial biogenesis in metabolically challenged Cardiomyocytes. Cells 10:3152. doi: 10.3390/cells10113152, PMID: 34831374 PMC8622724

[ref85] Melin F. Hellwig P. (2020). Redox properties of the membrane proteins from the respiratory chain. Chem. Rev. 120, 10244–10297. doi: 10.1021/acs.chemrev.0c00249 32820893

[ref86] Mi Y. Qi G. Vitali F. Shang Y. Raikes A. C. Wang T.. (2023). Loss of fatty acid degradation by astrocytic mitochondria triggers neuroinflammation and neurodegeneration. Nat. Metab. 5, 445–465. doi: 10.1038/s42255-023-00756-4, PMID: 36959514 PMC10202034

[ref87] Ming G. L. Song H. (2011). Adult neurogenesis in the mammalian brain: significant answers and significant questions. Neuron 70, 687–702. doi: 10.1016/j.neuron.2011.05.001, PMID: 21609825 PMC3106107

[ref88] Morant-Ferrando B. Jimenez-Blasco D. Alonso-Batan P. Agulla J. Lapresa R. Garcia-Rodriguez D.. (2023). Fatty acid oxidation organizes mitochondrial supercomplexes to sustain astrocytic ROS and cognition. Nat. Metab. 5, 1290–1302. doi: 10.1038/s42255-023-00835-6, PMID: 37460843 PMC10447235

[ref89] Moreno-Jiménez E. P. Flor-García M. Terreros-Roncal J. Rábano A. Cafini F. Pallas-Bazarra N.. (2019). Adult hippocampal neurogenesis is abundant in neurologically healthy subjects and drops sharply in patients with Alzheimer's disease. Nat. Med. 25, 554–560. doi: 10.1038/s41591-019-0375-9, PMID: 30911133

[ref90] Morita M. Ikeshima-Kataoka H. Kreft M. Vardjan N. Zorec R. Noda M. (2019). Metabolic plasticity of astrocytes and aging of the brain. Int. J. Mol. Sci. 20:941. doi: 10.3390/ijms20040941, PMID: 30795555 PMC6413111

[ref91] Morrison V. Houpert M. Trapani J. Brockman A. Kingsley P. Katdare K.. (2023). Jedi-1/MEGF12-mediated phagocytosis controls the pro-neurogenic properties of microglia in the ventricular-subventricular zone. Cell Rep. 42:113423. doi: 10.1016/j.celrep.2023.113423, PMID: 37952151 PMC10842823

[ref92] Nagao M. Ogata T. Sawada Y. Gotoh Y. (2016). Zbtb20 promotes astrocytogenesis during neocortical development. Nat. Commun. 7:11102. doi: 10.1038/ncomms11102, PMID: 27000654 PMC4804180

[ref93] Nardou R. Lewis E. M. Rothhaas R. Xu R. Yang A. Boyden E.. (2019). Oxytocin-dependent reopening of a social reward learning critical period with MDMA. Nature 569, 116–120. doi: 10.1038/s41586-019-1075-9, PMID: 30944474

[ref94] Obernier K. Alvarez-Buylla A. (2019). Neural stem cells: origin, heterogeneity and regulation in the adult mammalian brain. Development 146:dev156059. doi: 10.1242/dev.156059, PMID: 30777863 PMC6398449

[ref95] Ortiz-González X. R. (2021). Mitochondrial dysfunction: a common denominator in neurodevelopmental disorders? Dev. Neurosci. 43, 222–229. doi: 10.1159/000517870, PMID: 34350863 PMC8440386

[ref96] Ozgen S. Krigman J. Zhang R. Sun N. (2022). Significance of mitochondrial activity in neurogenesis and neurodegenerative diseases. Neural Regen. Res. 17, 741–747. doi: 10.4103/1673-5374.322429 34472459 PMC8530128

[ref97] Parousis A. Carter H. N. Tran C. Erlich A. T. Mesbah Moosavi Z. S. Pauly M.. (2018). Contractile activity attenuates autophagy suppression and reverses mitochondrial defects in skeletal muscle cells. Autophagy 14, 1886–1897. doi: 10.1080/15548627.2018.1491488, PMID: 30078345 PMC6152519

[ref98] Pecchillo Cimmino T. Pagano E. Stornaiuolo M. Esposito G. Ammendola R. Cattaneo F. (2022). Formyl-peptide receptor 2 signaling redirects glucose and glutamine into anabolic pathways in metabolic reprogramming of lung Cancer cells. Antioxidants (Basel) 11:1692. doi: 10.3390/antiox11091692, PMID: 36139766 PMC9495820

[ref99] Polis B. Srikanth K. D. Gurevich V. Bloch N. Gil-Henn H. Samson A. O. (2020). Arginase inhibition supports survival and differentiation of neuronal precursors in adult Alzheimer's disease mice. Int. J. Mol. Sci. 21:1133. doi: 10.3390/ijms21031133 32046281 PMC7037054

[ref100] Preez A. D. Onorato D. Eiben I. Musaelyan K. Egeland M. Zunszain P.. (2021). Chronic stress followed by social isolation promotes depressive-like behaviour, alters microglial and astrocyte biology and reduces hippocampal neurogenesis in male mice. Brain Behav. Immun. 91, 24–47. doi: 10.1016/j.bbi.2020.07.015, PMID: 32755644

[ref101] Preman P. Alfonso-Triguero M. Alberdi E. Verkhratsky A. Arranz A. M. (2021). Astrocytes in Alzheimer’s disease: pathological significance and molecular pathways. Cells 10:540. doi: 10.3390/cells10030540, PMID: 33806259 PMC7999452

[ref102] Qu Q. Wang J. Li G. Chen R. Qu S. (2021). The Conformationally sensitive spatial distance between the TM3-4 loop and transmembrane segment 7 in the glutamate transporter revealed by paired-cysteine mutagenesis. Front. Cell Dev. Biol. 9:737629. doi: 10.3389/fcell.2021.737629, PMID: 34621751 PMC8490817

[ref103] Quinzii C. M. Lopez L. C. (2021). Abnormalities of hydrogen sulfide and glutathione pathways in mitochondrial dysfunction. J. Adv. Res. 27, 79–84. doi: 10.1016/j.jare.2020.04.002, PMID: 33318868 PMC7728579

[ref104] Reichenbach B. Classon J. Aida T. Tanaka K. Genander M. Göritz C. (2018). Glutamate transporter Slc1a3 mediates inter-niche stem cell activation during skin growth. EMBO J. 37:e98280. doi: 10.15252/embj.201798280, PMID: 29615452 PMC5920238

[ref105] Ren Y. Z. Zhang B. Z. Zhao X. J. Zhang Z. Y. (2020). Resolvin D1 ameliorates cognitive impairment following traumatic brain injury via protecting astrocytic mitochondria. J. Neurochem. 154, 530–546. doi: 10.1111/jnc.14962, PMID: 31951012

[ref106] Rose J. Brian C. Pappa A. Panayiotidis M. I. Franco R. (2020). Mitochondrial metabolism in astrocytes regulates brain bioenergetics, neurotransmission and redox balance. Front. Neurosci. 14:536682. doi: 10.3389/fnins.2020.536682, PMID: 33224019 PMC7674659

[ref107] Rossi D. J. Jamieson C. H. Weissman I. L. (2008). Stems cells and the pathways to aging and cancer. Cell 132, 681–696. doi: 10.1016/j.cell.2008.01.036 18295583

[ref108] Roy D. Tedeschi A. (2021). The role of lipids, lipid metabolism and ectopic lipid accumulation in axon growth, regeneration and repair after CNS injury and disease. Cells 10:1078. doi: 10.3390/cells10051078, PMID: 34062747 PMC8147289

[ref109] Rubio-Atonal L. F. Ioannou M. S. (2023). Astrocytic OxPhos: more than just energy production. Nat. Metab. 5, 362–363. doi: 10.1038/s42255-023-00755-5 36959513

[ref110] Rubio-Casillas A. Fernández-Guasti A. (2016). The dose makes the poison: from glutamate-mediated neurogenesis to neuronal atrophy and depression. Rev. Neurosci. 27, 599–622. doi: 10.1515/revneuro-2015-0066, PMID: 27096778

[ref111] Santos R. Vadodaria K. C. Jaeger B. N. Mei A. Lefcochilos-Fogelquist S. Mendes A. P. D.. (2017). Differentiation of inflammation-responsive astrocytes from glial progenitors generated from human induced pluripotent stem cells. Stem Cell Rep. 8, 1757–1769. doi: 10.1016/j.stemcr.2017.05.011, PMID: 28591655 PMC5470172

[ref112] Sardar Sinha M. Ansell-Schultz A. Civitelli L. Hildesjö C. Larsson M. Lannfelt L.. (2018). Alzheimer's disease pathology propagation by exosomes containing toxic amyloid-beta oligomers. Acta Neuropathol. 136, 41–56. doi: 10.1007/s00401-018-1868-1, PMID: 29934873 PMC6015111

[ref113] Satarker S. Bojja S. L. Gurram P. C. Mudgal J. Arora D. Nampoothiri M. (2022). Astrocytic glutamatergic transmission and its implications in neurodegenerative disorders. Cells 11:1139. doi: 10.3390/cells11071139, PMID: 35406702 PMC8997779

[ref114] Schmukler E. Solomon S. Simonovitch S. Goldshmit Y. Wolfson E. Michaelson D. M.. (2020). Altered mitochondrial dynamics and function in APOE4-expressing astrocytes. Cell Death Dis. 11:578. doi: 10.1038/s41419-020-02776-4 32709881 PMC7382473

[ref115] Semple B. D. Blomgren K. Gimlin K. Ferriero D. M. Noble-Haeusslein L. J. (2013). Brain development in rodents and humans: identifying benchmarks of maturation and vulnerability to injury across species. Prog. Neurobiol. 106-107, 1–16. doi: 10.1016/j.pneurobio.2013.04.001, PMID: 23583307 PMC3737272

[ref116] Serrat R. Covelo A. Kouskoff V. Delcasso S. Ruiz-Calvo A. Chenouard N.. (2021). Astroglial ER-mitochondria calcium transfer mediates endocannabinoid-dependent synaptic integration. Cell Rep. 37:110133. doi: 10.1016/j.celrep.2021.110133 34936875

[ref117] Shu X. Sun Y. Sun X. Zhou Y. Bian Y. Shu Z.. (2019). The effect of fluoxetine on astrocyte autophagy flux and injured mitochondria clearance in a mouse model of depression. Cell Death Dis. 10:577. doi: 10.1038/s41419-019-1813-9, PMID: 31371719 PMC6675792

[ref118] Sies H. Mailloux R. J. Jakob U. (2024). Fundamentals of redox regulation in biology. Nat. Rev. Mol. Cell Biol. 25, 701–719. doi: 10.1038/s41580-024-00730-2 38689066 PMC11921270

[ref119] Sorrells S. F. Paredes M. F. Cebrian-Silla A. Sandoval K. Qi D. Kelley K. W.. (2018). Human hippocampal neurogenesis drops sharply in children to undetectable levels in adults. Nature 555, 377–381. doi: 10.1038/nature25975, PMID: 29513649 PMC6179355

[ref120] Stephen T. L. Higgs N. F. Sheehan D. F. Al Awabdh S. López-Doménech G. Arancibia-Carcamo I. L.. (2015). Miro1 regulates activity-driven positioning of mitochondria within astrocytic processes apposed to synapses to regulate intracellular calcium signaling. J. Neurosci. 35, 15996–16011. doi: 10.1523/JNEUROSCI.2068-15.2015, PMID: 26631479 PMC4666922

[ref121] Stoll E. A. Makin R. Sweet I. R. Trevelyan A. J. Miwa S. Horner P. J.. (2015). Neural stem cells in the adult subventricular zone oxidize fatty acids to produce energy and support neurogenic activity. Stem Cells 33, 2306–2319. doi: 10.1002/stem.2042, PMID: 25919237 PMC4478223

[ref122] Sulimai N. Brown J. Lominadze D. (2021). Fibrinogen interaction with astrocyte ICAM-1 and PrP(C) results in the generation of ROS and Neuronal death. Int. J. Mol. Sci. 22:2391. doi: 10.3390/ijms22052391, PMID: 33673626 PMC7957521

[ref123] Sun J. Li J. Y. Zhang L. Q. Li D. Y. Wu J. Y. Gao S. J.. (2021). Nrf2 activation attenuates chronic constriction injury-induced neuropathic pain via induction of PGC-1α-mediated mitochondrial biogenesis in the spinal cord. Oxidative Med. Cell. Longev. 2021:9577874. doi: 10.1155/2021/9577874, PMID: 34721761 PMC8554522

[ref124] Sun Y. Sukumaran P. Selvaraj S. Cilz N. I. Schaar A. Lei S.. (2018). TRPM2 promotes neurotoxin MPP+/MPTP-induced cell death. Mol. Neurobiol. 55, 409–420. doi: 10.1007/s12035-016-0338-9, PMID: 27957685 PMC5468501

[ref125] Suomalainen A. Nunnari J. (2024). Mitochondria at the crossroads of health and disease. Cell 187, 2601–2627. doi: 10.1016/j.cell.2024.04.037 38788685

[ref126] Szabo I. Szewczyk A. (2023). Mitochondrial ion channels. Annu. Rev. Biophys. 52, 229–254. doi: 10.1146/annurev-biophys-092622-094853 37159294

[ref127] Tao S. C. Yuan T. Rui B. Y. Zhu Z. Z. Guo S. C. Zhang C. Q. (2017). Exosomes derived from human platelet-rich plasma prevent apoptosis induced by glucocorticoid-associated endoplasmic reticulum stress in rat osteonecrosis of the femoral head via the Akt/bad/Bcl-2 signal pathway. Theranostics 7, 733–750. doi: 10.7150/thno.17450, PMID: 28255363 PMC5327646

[ref128] Toth A. B. Shum A. K. Prakriya M. (2016). Regulation of neurogenesis by calcium signaling. Cell Calcium 59, 124–134. doi: 10.1016/j.ceca.2016.02.011, PMID: 27020657 PMC5228525

[ref129] van den Berg N. W. E. Kawasaki M. Fabrizi B. Nariswari F. A. Verduijn A. C. Neefs J.. (2021). Epicardial and endothelial cell activation concurs with extracellular matrix remodeling in atrial fibrillation. Clin. Transl. Med. 11:e558. doi: 10.1002/ctm2.558, PMID: 34841686 PMC8567047

[ref130] Vasan K. Clutter M. Fernandez Dunne S. George M. D. Luan C. H. Chandel N. S.. (2022). Genes involved in maintaining mitochondrial membrane potential upon Electron transport chain disruption. Front. Cell Dev. Biol. 10:781558. doi: 10.3389/fcell.2022.781558, PMID: 35252167 PMC8888678

[ref131] Vasistha N. A. Pardo-Navarro M. Gasthaus J. Weijers D. Müller M. K. García-González D.. (2020). Maternal inflammation has a profound effect on cortical interneuron development in a stage and subtype-specific manner. Mol. Psychiatry 25, 2313–2329. doi: 10.1038/s41380-019-0539-5, PMID: 31595033 PMC7515848

[ref132] Vicente-Gutierrez C. Bonora N. Bobo-Jimenez V. Jimenez-Blasco D. Lopez-Fabuel I. Fernandez E.. (2019). Astrocytic mitochondrial ROS modulate brain metabolism and mouse behaviour. Nat. Metab. 1, 201–211. doi: 10.1038/s42255-018-0031-6, PMID: 32694785

[ref133] Vicente-Gutierrez C. Bonora N. Jimenez-Blasco D. Lopez-Fabuel I. Bates G. Murphy M. P.. (2021). Abrogating mitochondrial ROS in neurons or astrocytes reveals cell-specific impact on mouse behaviour. Redox Biol. 41:101917. doi: 10.1016/j.redox.2021.101917, PMID: 33711713 PMC7972977

[ref134] Wang P. Hao X. Li X. Yan Y. Tian W. Xiao L.. (2021). Curcumin inhibits adverse psychological stress-induced proliferation and invasion of glioma cells via down-regulating the ERK/MAPK pathway. J. Cell. Mol. Med. 25, 7190–7203. doi: 10.1111/jcmm.16749, PMID: 34169637 PMC8335680

[ref135] Wang L. Huang X. You X. Yi T. Lu B. Liu J.. (2020). Nanoparticle enhanced combination therapy for stem-like progenitors defined by single-cell transcriptomics in chemotherapy-resistant osteosarcoma. Signal Transduct. Target. Ther. 5:196. doi: 10.1038/s41392-020-00248-x, PMID: 32973147 PMC7518281

[ref136] Wang Y. Ni J. Gao T. Gao C. Guo L. Yin X. (2020). Activation of astrocytic sigma-1 receptor exerts antidepressant-like effect via facilitating CD38-driven mitochondria transfer. Glia 68, 2415–2426. doi: 10.1002/glia.23850, PMID: 32460411

[ref137] Wang Y. Ni J. Gao C. Xie L. Zhai L. Cui G.. (2019). Mitochondrial transplantation attenuates lipopolysaccharide- induced depression-like behaviors. Prog. Neuro-Psychopharmacol. Biol. Psychiatry 93, 240–249. doi: 10.1016/j.pnpbp.2019.04.010, PMID: 31022424

[ref138] Wegner M. S. Schömel N. Olzomer E. M. Trautmann S. Olesch C. Byrne F. L.. (2021). Increased glucosylceramide production leads to decreased cell energy metabolism and lowered tumor marker expression in non-cancerous liver cells. Cell. Mol. Life Sci. 78, 7025–7041. doi: 10.1007/s00018-021-03958-9, PMID: 34626204 PMC8558193

[ref139] Wu N. Sun X. Zhou C. Yan J. Cheng C. (2023). Neuroblasts migration under control of reactive astrocyte-derived BDNF: a promising therapy in late neurogenesis after traumatic brain injury. Stem Cell Res Ther 14:2. doi: 10.1186/s13287-022-03232-0, PMID: 36600294 PMC9814466

[ref140] Xu M. Guo Y. Wang M. J. Luo X. Shen X. Li Z.. (2023). L-arginine homeostasis governs adult neural stem cell activation by modulating energy metabolism in vivo. EMBO J. 42:e112647. doi: 10.15252/embj.2022112647, PMID: 36740997 PMC10015378

[ref141] Yang J. Yin M. Hou Y. Li H. Guo Y. Yu H.. (2022). Role of ammonia for brain abnormal protein glycosylation during the development of hepatitis B virus-related liver diseases. Cell Biosci. 12:16. doi: 10.1186/s13578-022-00751-4, PMID: 35164881 PMC8842931

[ref142] Zamboni M. Llorens-Bobadilla E. Magnusson J. P. Frisén J. (2020). A widespread neurogenic potential of neocortical astrocytes is induced by injury. Cell Stem Cell 27, 605–617.e5. doi: 10.1016/j.stem.2020.07.006, PMID: 32758425 PMC7534841

[ref143] Zehnder T. Petrelli F. Romanos J. De Oliveira Figueiredo E. C. Lewis T. L. Jr. Déglon N.. (2021). Mitochondrial biogenesis in developing astrocytes regulates astrocyte maturation and synapse formation. Cell Rep. 35:108952. doi: 10.1016/j.celrep.2021.108952, PMID: 33852851

[ref144] Zhang Z. Chen H. Geng Z. Yu Z. Li H. Dong Y.. (2022). Structural basis of ligand binding modes of human EAAT2. Nat. Commun. 13:3329. doi: 10.1038/s41467-022-31031-x, PMID: 35680945 PMC9184463

[ref145] Zhang Y. M. Qi Y. B. Gao Y. N. Chen W. G. Zhou T. Zang Y.. (2023). Astrocyte metabolism and signaling pathways in the CNS. Front. Neurosci. 17:1217451. doi: 10.3389/fnins.2023.1217451, PMID: 37732313 PMC10507181

[ref146] Zhang T. Zhang Y. Liu J. Ma Y. Ye Q. Yan X.. (2022). MicroRNA-377-3p inhibits hepatocellular carcinoma growth and metastasis through negative regulation of CPT1C-mediated fatty acid oxidation. Cancer Metab. 10:2. doi: 10.1186/s40170-021-00276-3, PMID: 35057851 PMC8772112

[ref147] Zhang Z. Zheng X. Luan Y. Liu Y. Li X. Liu C.. (2018). Activity of metabotropic glutamate receptor 4 suppresses proliferation and promotes apoptosis with inhibition of Gli-1 in human glioblastoma cells. Front. Neurosci. 12:320. doi: 10.3389/fnins.2018.00320, PMID: 29867331 PMC5962807

[ref148] Zhang X. Zhu L. B. He J. H. Zhang H. Q. Ji S. Y. Zhang C. N.. (2020). Paroxetine suppresses reactive microglia-mediated but not lipopolysaccharide-induced inflammatory responses in primary astrocytes. J. Neuroinflammation 17:50. doi: 10.1186/s12974-020-1712-0, PMID: 32024542 PMC7003432

[ref149] Zhao J. Qu D. Xi Z. Huan Y. Zhang K. Yu C.. (2021). Mitochondria transplantation protects traumatic brain injury via promoting neuronal survival and astrocytic BDNF. Transl. Res. 235, 102–114. doi: 10.1016/j.trsl.2021.03.017 33798765

[ref150] Zheng Q. Chen X. Qiao C. Wang M. Chen W. Luan X.. (2021). Somatic CG6015 mediates cyst stem cell maintenance and germline stem cell differentiation via EGFR signaling in Drosophila testes. Cell Death Discov. 7:68. doi: 10.1038/s41420-021-00452-w, PMID: 33824283 PMC8024382

[ref151] Zhou W. Zhao T. Du J. Ji G. Li X. Ji S.. (2019). TIGAR promotes neural stem cell differentiation through acetyl-CoA-mediated histone acetylation. Cell Death Dis. 10:198. doi: 10.1038/s41419-019-1434-3 30814486 PMC6393469

